# When Russia Takes Any Matter in Hand

**Published:** 1899-10

**Authors:** 


When Russia takes any matter in hand it is generally carried out in a thorough manner. I see that the Russian Public Health Society has appointed a committee, under the presidency of Dr. Limberg, for the organization of a regular system of dental inspection and treatment in schools not only in St. Petersburg, but throughout the empire. As a preliminary step the committee has sent out a circular of questions to all heads of schools of the lower and middle grades, and to all practitioners having medical charge of such schools. It will be interesting, if these results are published, to compare them with the similar work which has been carried on for some years in some of our Board of Guardians' Schools, and by the lately-formed School of Dentists' Society.—*The Dental Record.*

